# The effect of metronidazole plus amoxicillin or metronidazole plus penicillin V on a periodontal biofilm model. An *in vitro s*tudy.

**DOI:** 10.1080/20002297.2017.1325270

**Published:** 2017-06-01

**Authors:** Sundus Saad Al-Zubaydi, Gabriela Dabija-Wolter, Marwan Mansoor Ali Mohammed, Anne Isine Bolstad

**Affiliations:** ^a^ Faculty of Medicine and Dentistry, Department of Clinical Dentistry- Periodontics, University of Bergen, Bergen, Norway; ^b^ Faculty of Medicine and Dentistry, Department of Clinical Science, University of Bergen, Bergen, Norway

## Abstract

**Background:** Antibiotic resistance has become life-threatening. A combination of metronidazole and amoxicillin is commonly used as adjunct to periodontal therapy. The aim of this study was to utilize an *in vitro* biofilm model to test the effect of replacing the broad-spectrum amoxicillin with penicillin V in the combination with metronidazole.

**Methods:** An *in vitro* biofilm model, consisting of *Aggregatibacter actinomycetemcomitans, Porphyromonas gingivalis* and *Fusobacterium nucleatum* was developed. Biofilms were exposed to chlorhexidine digluconate (CHX) 0.2%, as positive control, and either to metronidazole/amoxicillin or metronidazole/penicillin V in two different concentrations. Statistical significance was achieved if p < 0.05 with paired t-test.

**Results:** There was no growth of any bacterial species after 2h exposure to 0.2% CHX. Together, the two combinations of antibiotics were effective against *F. nucleatum* and *P. gingivalis*, but less effective against *A. actinomycetemcomitans*. No significant difference was found between the two combinations of antibiotics to combat *A. actinomycetemcomitans* neither in high (p = 0.95) nor in low concentration (p = 0.17).

**Conclusion:** A combination of metronidazole with penicillin V had similar inhibiting effect as metronidazole combined with amoxicillin on an *in vitro* three-species biofilm model. Use of a narrow spectrum antibiotic may be indicated in combination with metronidazole.

